# 5-Repurposed Drug Candidates Identified in Motor Neurons and Muscle Tissues with Amyotrophic Lateral Sclerosis by Network Biology and Machine Learning Based on Gene Expression

**DOI:** 10.1007/s12017-025-08847-z

**Published:** 2025-04-03

**Authors:** Kubra Temiz, Aytac Gul, Esra Gov

**Affiliations:** 1 Department of Bioengineering, Faculty of Engineering, Adana Alparslan Türkeş Science and Technology University, Building M1, Office: 202 Saricam, 01250 Adana, Türkiye; 2 Present Address: Department of Biotechnology, Faculty of Science, Bartin University, Bartin, Türkiye; 3 Department of Medical Biology, Tayfur Ata Sökmen Faculty of Medicine, Hatay Mustafa Kemal University, Hatay, Türkiye

**Keywords:** Amyotrophic lateral sclerosis, Differential co-expression analysis, Drug repurposing, Machine learning, Text-mining

## Abstract

Amyotrophic lateral sclerosis (ALS) is a progressive neurodegenerative disorder that leads to motor neuron degeneration, muscle weakness, and respiratory failure. Despite ongoing research, effective treatments for ALS are limited. This study aimed to apply network biology and machine learning (ML) techniques to identify novel repurposed drug candidates for ALS. In this study, we conducted a meta-analysis using 4 transcriptome data in ALS patients (including motor neuron and muscle tissue) and healthy controls. Through this analysis, we uncovered common shared differentially expressed genes (DEGs) separately for motor neurons and muscle tissue. Using common DEGs as proxies, we identified two distinct clusters of highly clustered differential co-expressed cluster genes: the 'Muscle Tissue Cluster' for muscle tissue and the 'Motor Neuron Cluster' for motor neurons. We then evaluated the performance of the nodes of these two modules to distinguish between diseased and healthy states with ML algorithms: KNN, SVM, and Random Forest. Furthermore, we performed drug repurposing analysis and text-mining analyses, employing the nodes of clusters as drug targets to identify novel drug candidates for ALS. The potential impact of the drug candidates on the expression of cluster genes was predicted using linear regression, SVR, Random Forest, Gradient Boosting, and neural network algorithms. As a result, we identified five novel drug candidates for the treatment of ALS: Nilotinib, Trovafloxacin, Apratoxin A, Carboplatin, and Clinafloxacin. These findings highlight the potential of drug repurposing in ALS treatment and suggest that further validation through experimental studies could lead to new therapeutic avenues.

**Supplementary Information:**

The online version contains supplementary material available at 10.1007/s12017-025-08847-z.

## Significance Statement

Amyotrophic lateral sclerosis (ALS) is a severe neurodegenerative disease characterized by progressive loss of motor neurons, leading to muscle weakness and paralysis, with limited treatment options. This study was carried out to address the urgent need for ALS therapies by leveraging network biology and machine learning. We identified key gene clusters that distinguish healthy from diseased states by analyzing gene expression in motor neurons and muscle tissues. Through drug repurposing analysis, we identified five promising therapeutic candidates for ALS: Nilotinib, Trovafloxacin, Apratoxin A, Carboplatin, and Clinafloxacin. These findings provide a novel, data-driven approach to ALS drug discovery, offering valuable insights into disease mechanisms that could shape future therapeutic strategies and inspire further experimental research.

## Introduction

Amyotrophic lateral sclerosis (ALS) is a fatal neurodegenerative disorder characterized by the progressive degeneration of motor neurons, both upper motor neurons located in the brain and lower motor neurons within the spinal cord and peripheral nerves. This degeneration leads to severe clinical manifestations, including muscle weakness, atrophy, and, ultimately, respiratory failure (Petri et al., 2023) Despite extensive research, the underlying etiology of ALS remains largely unknown, although approximately 5–10% of cases are familial, with mutations in genes such as *C9orf72*, *TARDBP*, *FUS*, and *SOD1* implicated in its pathogenesis (Prasad et al., 2019). Several molecular mechanisms have been proposed to contribute to ALS, including glutamate excitotoxicity (Upadhya et al., 2020), oxidative stress, mitochondrial dysfunction, and protein misfolding (Kodavati et al., 2020). However, despite advancements in understanding the disease mechanism, treatment options remain limited. While drugs like Riluzole, Edaravone (Jiang et al., 2022), and Tofersen (Miller et al., 2022) have shown some capacity to slow disease progression, there remains no definitive cure for ALS (Jiang et al., 2022). Therefore, there is a critical need for novel therapeutic strategies and a deeper understanding of ALS’s molecular basis.

Network biology offers a powerful, integrative approach to investigating complex biological systems, allowing researchers to infer molecular, genetic, and physiological interactions within an organism. This systems-oriented computational perspective facilitates the integration of experimental and clinical data, providing a holistic view of disease processes (Wang, 2022). By employing computational techniques to construct networks of biological interactions, network biology helps overcome the limitations of individual experimental studies, particularly in diseases like ALS, where the underlying molecular mechanisms are intricate and multifactorial. Specifically, network-based approaches such as protein–protein interaction (PPI) networks and differential co-expression analyses enable the identification of significant gene clusters and biomolecules associated with the disease. This framework has proven valuable for discovering disease biomarkers and potential therapeutic targets, particularly in diseases with complex pathophysiology such as ALS (Comte et al., 2020; Fiscon et al., 2018).

In parallel, drug repurposing has emerged as a promising strategy to ease and accelerate the development of therapeutic interventions. Drug repurposing refers to the identification of new therapeutic uses for existing drugs that have already been approved for other indications. This approach significantly reduces both the time and cost associated with drug development, as the pharmacokinetics, pharmacodynamics, and safety profiles of these drugs are already well established (Kori et al., 2023; Pushpakom et al., 2019). In contrast to traditional drug discovery, which can take over a decade and requires substantial financial investment, repurposing allows researchers to bypass early-stage clinical trials, focusing instead on phase-2 and phase-3 studies. This strategy has demonstrated success in the treatment of various diseases and holds considerable potential in ALS, where effective treatment options are critically lacking (Hua et al., 2022).

**Fig. 1:**
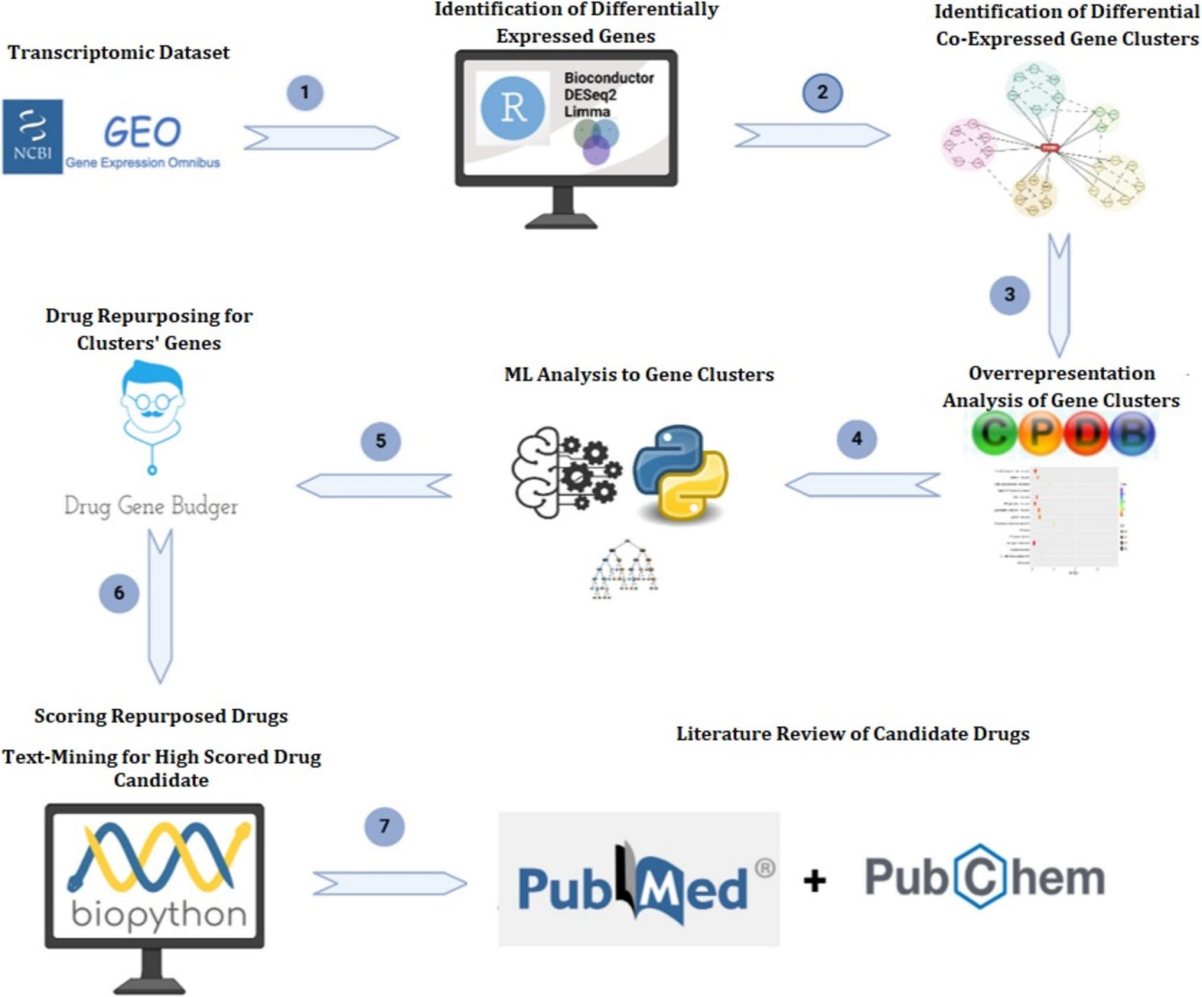
The computational workflow utilized in this study

In this study, we applied transcriptomic analyses of muscle tissue and motor neurons from ALS patients using high-throughput sequencing and expression profiling techniques. Meta-analysis of these datasets allowed for the identification of gene pairs exhibiting differential co-expression between ALS patients and controls. We then used network biology to create co-expression networks and identify key clusters within these networks. These clusters were evaluated by machine learning (ML) algorithms for their accuracy in distinguishing between healthy and diseased states. The nodes of modules selected according to a set threshold were used as proxies to identify potential drug candidates. Five different ML algorithms were applied to evaluate the linear and non-linear effects of candidate drugs on the expression of module genes: Linear Regression, Support Vector Regression (SVR), Random Forest Regression, Gradient Boost Regression, and Neural Network. To assess the novelty potential of these candidates, text-mining was performed and their physical interactions with key gene clusters were examined using the PubChem database (Fig. 1).

## Materials and Methods

### Gene Expression Datasets

In order to examine the transcriptomic effects of ALS, a neuromuscular disease, in comparison to healthy controls, a detailed search was conducted in the NCBI Gene Expression Omnibus (GEO) database (Barrett et al., 2013). In this context, the keywords 'Amyotrophic Lateral Sclerosis,' 'Muscle Tissue,' and 'Motor Neuron Tissue' were used. Within the scope of this research, only studies involving the *Homo sapiens* organism and comparing ALS patients with healthy control groups were selected. The datasets selected to analyze the effects of ALS on gene expression focused on motor neurons and muscle tissue. For motor neuron tissue, an RNA-seq dataset (GSE76220, 8 healthy, 13 ALS samples) (Krach et al., 2018) and a gene expression dataset (GSE19332, 7 healthy, 3 ALS samples) (Cox et al., 2010) were used. For muscle tissue, the gene expression datasets GSE26276 (3 ALS, 3 control) (Shtilbans et al., 2011) and GSE41414 (7 ALS, 7 control) (Bernardini et al., 2013) were utilized.

### Identification of Differentially Expressed Genes

A robust statistical analysis pipeline was employed to identify differentially expressed genes (DEGs) across the microarray datasets. Raw data were normalized using the Robust Multi-Array Average (RMA) method (Bolstad et al., 2003), as implemented in the Affy package (Gautier et al., 2004) of the R/Bioconductor platform (Huber et al., 2015). DEG identification was performed using the Linear Models for Microarray Data (LIMMA) package (Ritchie et al., 2015), which is widely used for its sensitivity and flexibility in analyzing microarray data. For RNA-seq datasets, the raw data were processed with DESeq2 (Love et al., 2014), which provides accurate identification of DEGs in count-based RNA-seq data. For microarray datasets, we utilized the Robust Multi-Array Average (RMA) method for normalization, which is highly effective in reducing technical variability. For RNA-seq data, DESeq2 employs internal normalization using the median of ratios method, which accounts for differences in library size and composition. These normalization steps ensure data comparability and minimize technical artifacts. The Benjamini–Hochberg method was applied to control the false discovery rate (FDR), with an adjusted p value of < 0.05 used to define statistical significance. DEGs were further filtered based on fold change thresholds, with values greater than 1.5 indicating upregulation and less than 0.67 indicating downregulation. To identify shared molecular signatures, the results from both microarray and RNA-seq analyses were comparatively assessed and overlapping DEGs were selected for downstream analyses.

### Co-Expression Network Analysis

Co-expression analysis, an established method for studying gene correlations (Gov & Arga, 2017), was performed on common DEGs identified from RNA-seq data, which are shared between disease and control states, to evaluate the relationships between gene pairs. The expression levels of the DEGs were normalized using z-score transformation to standardize the data across samples. Pearson Correlation Coefficient (PCC) was used for gene pairs with a normal distribution, while Spearman Correlation Coefficient (SCC) was applied for non-normally distributed gene pairs. A p value threshold of < 0.05 was applied to determine statistically significant pairwise correlations. Co-expression networks were constructed based on these significant correlations, capturing gene–gene interactions in both disease and healthy samples. The networks were then visualized using Cytoscape (version 3.10.2) (Shannon et al., 2003) for a better interpretation of gene interactions.

### Identification of Differential Co-expression Network Analysis and Clusters

Significantly co-expressed genes were evaluated by computing p-critical values for common differentially expressed genes (DEGs), based on pairwise gene expression correlation values using Spearman's Correlation Coefficients (SCCs). The *p*-critical value (*P*_critic_), which represents the threshold for identifying significant correlations, was calculated as follows:

1 $$p_{{{\text{critic}}}} = {\text{meanSCC}} + 1.96{\text{*stdofSCC}}$$

This value helps filter out weak correlations, focusing only on gene pairs with SCC values that exceed this threshold, thereby capturing more robust gene–gene interactions.

To identify gene pairs with significant associations between disease and healthy states, a threshold parameter *ε* was applied. The threshold condition was defined as

2 $$\varepsilon = \left| {{\text{SCCd}} - {\text{SCCh}}} \right| > 0.5,$$

where SCCd and SCCh represent Spearman's Correlation Coefficients in the disease and healthy states, respectively. The threshold of 0.5 was chosen to capture meaningful differences in gene expression correlations, ensuring that only gene pairs with biologically significant changes between the two conditions were included. These thresholds were crucial in constructing co-expression networks, allowing the identification of key gene interactions and potential regulatory mechanisms relevant to the disease process.

In the network analysis, edges represent significant gene–gene correlations based on SCCs. These correlations capture expression relationships between genes in disease and healthy states, highlighting biologically relevant changes (Gov & Arga, 2017). The network does not depict physical interactions like PPI networks but rather dynamic co-expression patterns. Network density assesses the level of interconnectivity within a network, offering valuable insights into its overall framework (Silva et al., 2018). Network density is calculated as

$$D = \frac{2E}{{V\left( {V - 1} \right)}},$$

where *E* = Number of edges, *V* = Number of nodes.

Molecular Complex Detection Score (MCODE score) evaluates how well genes form distinct functional modules (Bader & Hogue, 2003). Higher modularity suggests well-defined gene clusters, which indicate coordinated biological processes.

### Enrichment Analysis of Differentially Co-expressed Gene Clusters

Pathway-based overrepresentation analyses were conducted using ConsensusPathDB (Kamburov et al., 2009) to identify enriched biological pathways of the significant clusters. The Kyoto Encyclopedia of Genes and Genomes (KEGG) (Kanehisa & Goto, 2000) and Reactome (Fabregat et al., 2018) databases were used as pathway references. Pathways with an adjusted *p* value < 0.05, corrected using the Benjamini–Hochberg method to control the FDR were considered statistically significant. Analyses were performed separately for both KEGG and Reactome databases to ensure comprehensive pathway identification.

### Machine Learning Analysis of Differential Co-expressed Cluster for Validation Analysis

In this study, the GSE234297 dataset (Grima et al., 2023) was used. The dataset contains 48 healthy and 96 ALS samples. Gene expression data were used to examine differences between healthy and ALS samples. Genes in the dataset were identified as differential expression networks of healthy and diseased samples and features were selected as gene expression values. CatBoost, XGBoost, Random Forest, K-Neighbors, LightGBM, Decision Tree, Gradient Boosting, MLP, and SVM algorithms were applied using the Python programming language. Gene expression data were modeled with 80% training and 20% testing split and evaluated using performance metrics such as accuracy, F1 score, and recall. In the data preprocessing stage, gene expression values were normalized using the StandardScaler method. Each model was trained separately for each gene using gene expression values as features to better understand the effect of genes on the models. The results were visualized with the matplotlib library and analyzed by comparing the classification success of the algorithms. This method allowed to evaluate the potential biomarker roles of genes belonging to motor neuron and muscle clusters in ALS diagnosis.

### Identification of Candidate Drugs by Using Machine Learning Algorithms

Drug repurposing analysis was conducted using Drug Gene Budger (DGB) (Wang et al., 2019), a tool designed to identify small molecules or drugs capable of modulating gene expression levels. DGB compiles experimental data from several databases, including NCBI GEO, L1000, and the original Connectivity Map (CMap) (Wang et al., 2019). In this study, the CRowd Extracted Expression of Differential Signatures (CREEDS), available on the DGB platform, was employed to determine whether drugs could reverse disease-associated gene expression changes to healthy-like levels by targeting key regulatory genes.

The analysis yielded several parameters, including "Drug name," "CREEDS ID," "GEO ID," "*p*-value," "*q*-value," "Drug fold change (FC)," and "Specificity," with statistical significance thresholds set at *p* value < 0.05 and *q*-value < 0.05. Drugs were selected based on a fold change criterion, where an FC greater than 1.5 indicated upregulation, and an FC less than 0.67 indicated downregulation. These thresholds were chosen to capture biologically significant gene expression changes that might influence disease pathways.

After identifying potential drug candidates through DGB, we applied several ML algorithms to further analyze the relationships between gene expression profiles and drug effects. Specifically, we employed Random Forest (RF) for its robustness and ability to handle high-dimensional data, Support Vector Machine (SVM) for capturing complex relationships via kernel functions, Logistic Regression (LR) as an interpretable baseline model, Gradient Boosting Machines (GBM) for modeling non-linear dependencies, and k-Nearest Neighbors (k-NN) as a non-parametric approach. These algorithms were chosen for their complementary strengths, and their performance was assessed using accuracy, precision, recall, F1 score, and AUC-ROC. To ensure optimal model performance, hyperparameters were fine-tuned using grid search with cross-validation. Input data were constructed from matrices containing fold changes, gene names, and drug names for each gene cluster. Categorical variables were created using One-Hot Encoding (Okada et al., 2019), and the independent variables (i.e., gene FCs and gene names) and target variable (i.e., drug FC) were identified. The data were normalized using the StandardScaler to ensure consistent feature scaling, thereby improving the performance of the ML models. Five different models were applied to the normalized data: Linear Regression, Support Vector Regression (SVR) (Cortes & Vapnik, 1995), Random Forest Regressor (Pal, 2005), Gradient Boosting Regressor (Friedman, 2001), (Waskom, 2021), and Neural Network (MLPRegressor) (Rumelhart et al., 1986). These models were selected to capture both linear and non-linear relationships between gene expression and drug effects, enabling comprehensive predictions about drug impact on gene expression. The performance of the models was evaluated using cross-validation, which provided robust estimates of predictive accuracy. Predictions from each model were stored in a DataFrame and exported to Excel for further analysis. Data visualizations were generated using Seaborn (Waskom, 2021) to illustrate model performance and predicted drug effects on gene expression.

### Text-Mining Analysis of Candidate Drugs

A text-mining approach was employed to assess the novelty of the candidate drugs to complement the ML predictions. Using Python, two different keyword sets—"drug candidate" and "drug candidate + target disease"—were used to search relevant article abstracts. This process was conducted using the BioPython package (Cock et al., 2009), and Term Frequency (TF) and Inverse Document Frequency (IDF) were calculated to assess how frequently each drug appeared in the literature relative to the target disease. TF-IDF is useful in this context as it highlights drugs that are rarely mentioned in the literature, making it an effective method for identifying novel drugs.

A TF-IDF score of 0 indicated that a drug had not been associated with the target disease in the literature, signifying it as a potential novel candidate. Finally, the physical interactions between the identified drug candidates and target genes were validated by querying the PubMed and PubChem databases. PubMed was used to identify relevant studies, while PubChem was queried to retrieve known drug–gene interaction data, ensuring that the proposed drug–gene associations were supported by experimental evidence.

## Results

### Gene Expression Patterns in ALS: Insights from Muscle Tissues and Motor Neurons

**Fig. 2:**
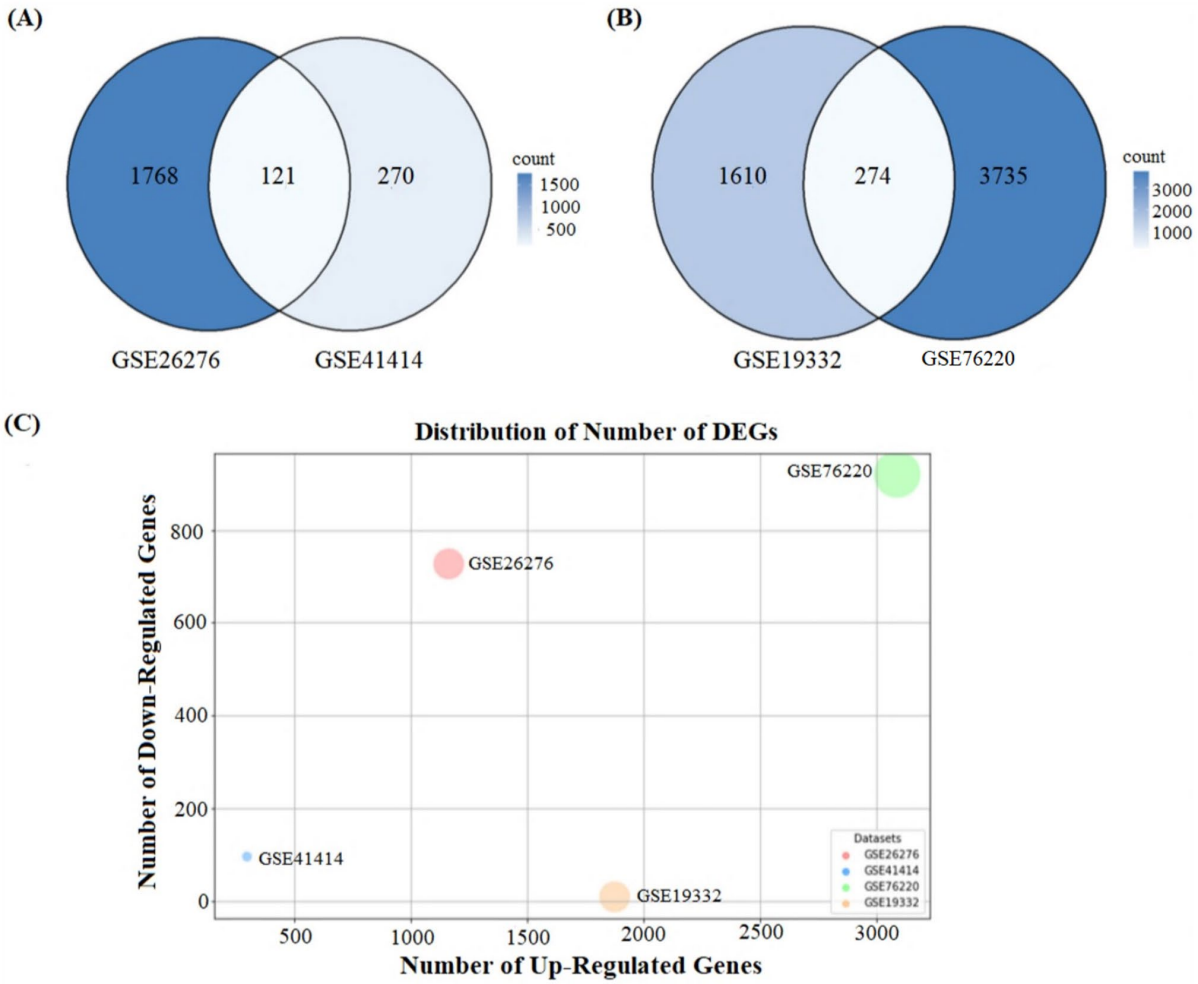
Venn diagram showing the common DEGs between transcriptome data. **A** Common DEGs muscle tissues of ALS. **B** Common DEGs motor neurons of ALS. **C** Distribution of DEGs across ALS datasets

The ALS gene expression data were categorized into two types of muscle tissue and motor neurons. A total of 121 common differentially expressed genes (DEGs) were identified in muscle tissue, while 274 common DEGs were obtained in motor neurons, as shown in Fig. 2A, B, illustrating a Venn diagram comparing DEGs in muscle tissues from two datasets, GSE26276 and GSE41414. The common DEGs were selected based on their presence in multiple datasets, without requiring consistent up- or downregulation across all datasets. In this comparison, 1768 DEGs are unique to GSE26276, 270 are unique to GSE41414, and 121 DEGs are shared between the two, indicating a consistent pattern of gene expression in muscle tissues across datasets. Similarly, Fig. 2B compares DEGs in motor neurons between GSE19332 and GSE76220, with 1610 DEGs unique to GSE19332, 3735 unique to GSE76220, and 274 common DEGs. This overlap suggests that a core set of DEGs in motor neurons is consistently identified across multiple datasets, reinforcing the robustness of the findings. Figure 2C provides a scatter plot showing the distribution of upregulated and downregulated genes across four datasets. The GSE76220 dataset exhibits the highest number of both upregulated and downregulated genes, followed by GSE19332 and GSE41414, while GSE26276 has the fewest DEGs overall. Among the DEGs identified, 63.93% were upregulated in muscle tissues, whereas 84% of DEGs were upregulated in motor neurons. These results suggest that gene expression changes in motor neurons are more pronounced compared to muscle tissues, a key finding in understanding ALS pathophysiology. However, it is important to note that the GSE76220 dataset has a larger sample size compared to the other datasets, which could potentially influence the number of identified DEGs. So it may cause a bias in the identification of DEGs. To mitigate this potential bias, common DEGs were used to minimize the bias and conducted multiple downstream analyses, including differential co-expression analysis and machine learning approaches, to validate and further explore the biological relevance of these findings.

### Differential Co-expressed Clusters and Their Biological Insights

**Fig. 3:**
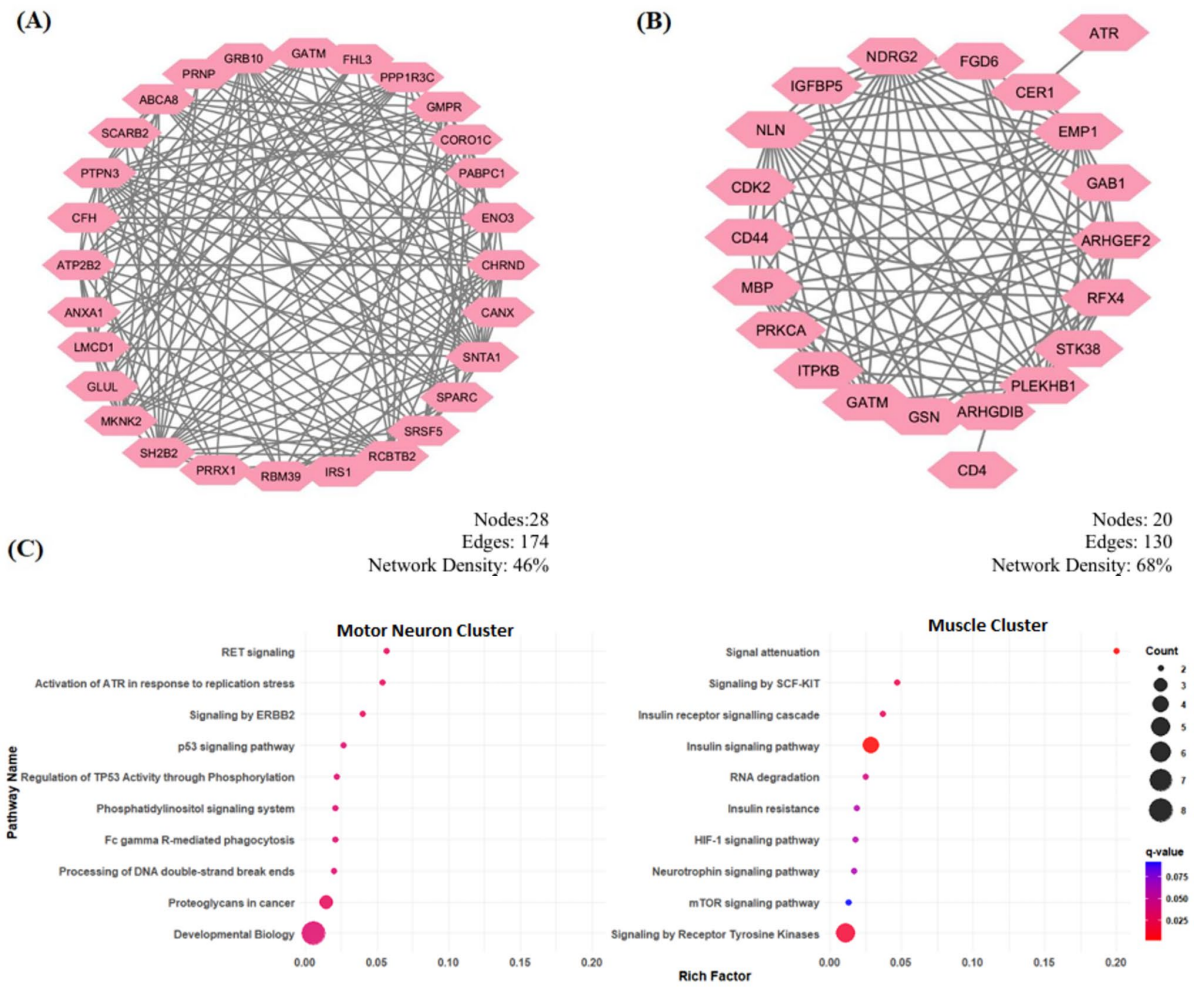
Co-expression network analysis and gene enrichment in ALS. **A** Differentially Co-expressed genes within the Muscle Cluster in ALS. **B** Differentially co-expressed genes within the Motor Neuron Cluster in ALS. **C** Gene enrichment analysis of Muscle Cluster, and Motor Neuron Cluster

After identifying common differentially expressed genes (DEGs) in both motor neurons and muscle tissue, differential co-expressed networks were constructed to investigate the relationships between these DEGs. Key differential co-expressed gene pairs were identified using Spearman correlation coefficients (SCCs), forming the basis for network construction. In muscle tissue, five clusters were initially identified; however, only one cluster met the significance threshold. The cluster, referred to as the ‘Muscle Tissue Cluster,’ comprises 28 nodes (genes) and 174 edges (co-expressed gene pairs), with a network density of 46% and an mCode score of 12.89 (Fig. 3A). For motor neurons, four clusters were identified, but only one cluster satisfied the significance criteria. The cluster, termed the ‘Motor Neuron Cluster,’ consists of 20 nodes and 130 edges, with a higher network density of 68% and an mCode score of 13.68 (Fig. 3B). Network density and modularity scores provide insights into the organization and connectivity of the co-expressed gene networks in motor neurons and muscle tissues. Higher network density indicates a more interconnected set of genes, while higher modularity scores suggest the presence of distinct, biologically meaningful clusters, which are essential for understanding the underlying mechanisms of ALS in these tissues. Pathway enrichment analysis for muscle tissue (Fig. 3C) revealed significant disruptions in metabolism and muscle function pathways, with the signal attenuation pathway being the most enriched with a q-value of 0.00225 and a rich factor of 0.2. The insulin-related pathway suggests metabolic disturbances, while pathways such as tyrosine kinase signaling, and RNA degradation indicate potential disruptions in muscle growth regulation and RNA processing. The high rich factor value for these pathways indicates a substantial involvement of differentially expressed genes (DEGs) in muscle dysfunction in ALS. In motor neurons (Fig. 3C), the pathways were involved in RET signaling and ATR activation in response to replication stress highlighting critical roles in neuronal survival and DNA damage response mechanisms. Additionally, the enrichment of the p53 signaling pathway and G2/M checkpoints suggests a link to cell cycle dysregulation and apoptosis, while neurotrophin signaling points to impaired neurotrophic support in ALS motor neuron degeneration.

### Machine Learning Analysis of Clusters and Clusters’ Genes

**Fig. 4:**
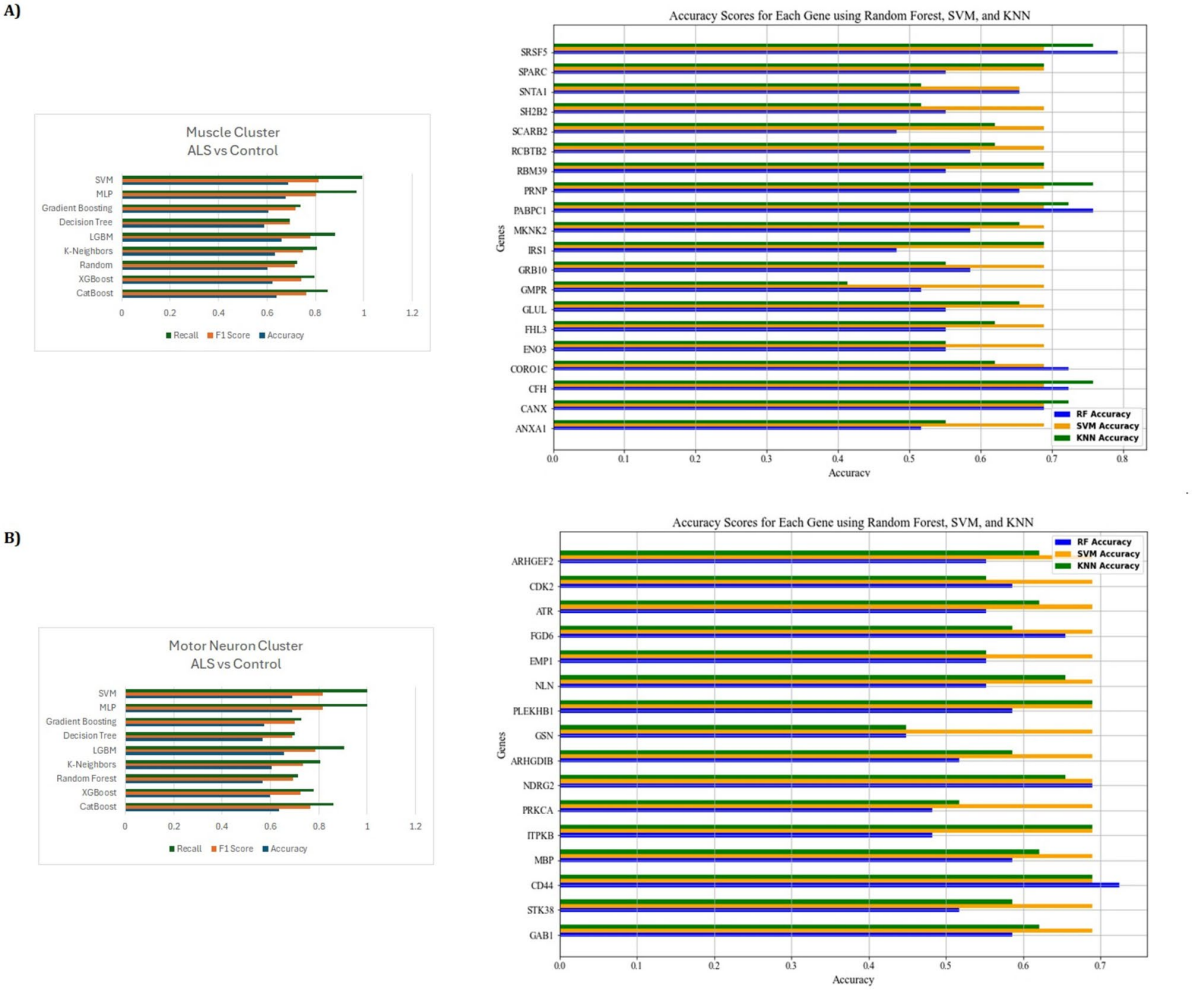
Machine Learning Analysis of Gene Clusters **A** Analysis of Genes in the Muscle Gene Cluster. **B** Analysis of Genes in the Motor Neuron Gene Cluster

We applied ML algorithms to the clusters to evaluate the performance of the dataset in classifying ALS versus control. In the ALS Motor Neuron Cluster, SVM and MLP models showed the highest performance in the classification of motor neuron clusters in ALS disease. The recall value of both models was perfectly measured as 1.0, while the accuracy and F1 score were 0.6897 and 0.8163 for SVM and MLP, respectively. LGBM (accuracy: 0.6573, recall: 0.9063) and CatBoost (accuracy: 0.6358, recall: 0.8594) also achieved very strong results. The performance of the other models remained at lower levels: XGBoost (accuracy: 0.5991, recall: 0.7781) and K-Neighbors (accuracy: 0.6056, recall: 0.8063) achieved lower results. Random Forest (accuracy: 0.5668, recall: 0.7156) and Decision Tree (accuracy: 0.5690, recall: 0.7) were ranked as the lowest performing models (Fig. 4A). The classification performance of individual motor neuron cluster genes is also remarkable. Genes such as SRSF5, PABPC1, CFH, CORO1C, CANX, and PRNP achieved classification scores above 0.6 in all models. In particular, SRSF5 achieved the highest score of 0.79 in the RF model, while the highest score of the PABPC1 gene was 0.76 in RF and the CFH gene was 0.72 in RF. This trend was similarly observed for the motor neuron gene set.

For the classification of muscle tissue, SVM (accuracy: 0.6879, recall: 0.995) and MLP (accuracy: 0.6776, recall: 0.97) models performed best with the highest recall values. LGBM (accuracy: 0.6603, recall: 0.8825) and CatBoost (accuracy: 0.6397, recall: 0.8525) gave good results. K-Neighbors (accuracy: 0.6345, recall: 0.805) and XGBoost (accuracy: 0.6241, recall: 0.795) performed worse. Random Forest (accuracy: 0.6034, recall: 0.725) and Decision Tree (accuracy: 0.5897, recall: 0.695) were the worst performing models (Fig. 4B). The classification performance of individual genes for the muscle cluster also provides important findings. The RF model achieved accuracy scores of 0.72 and 0.69 with CD44 and NDRG2 genes, respectively. These genes generally performed above 0.65 in all algorithms. However, genes such as GSN and PRKCA had lower accuracy scores, especially in the KNN and RF algorithms, with values as low as 0.45. The SVM model performed consistently, achieving accuracies up to 0.69 for most genes.

### Candidate Drugs for ALS

**Fig. 5:**
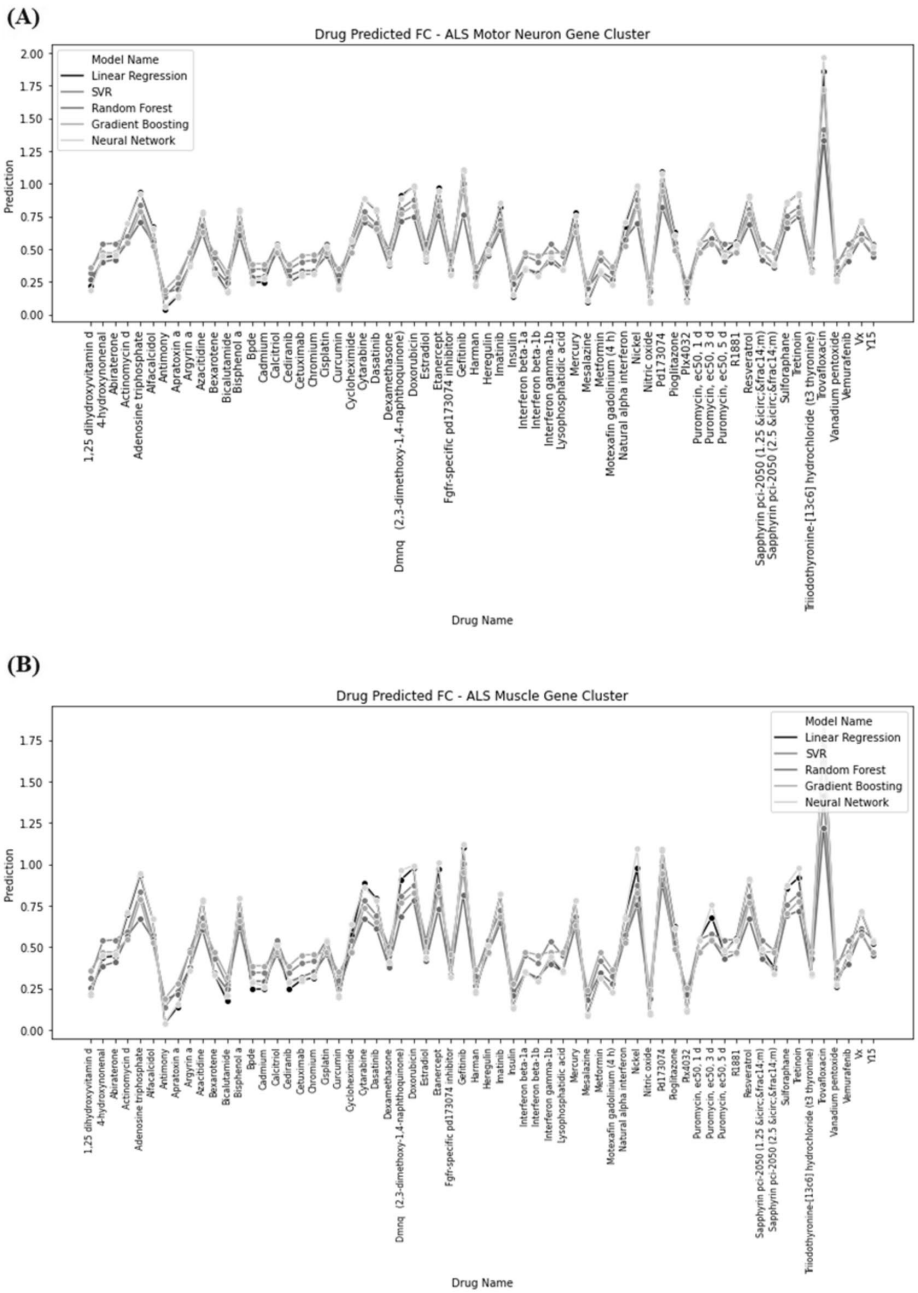
Machine Learning Analysis of Drug Repurposing Efforts. **A** Targeting Genes Within the Motor Neuron Cluster. **B** Drug Predicted FC targeting ALS Muscle Gene Cluster

We applied a drug repositioning approach using multiple ML models to evaluate drug candidates for the Motor Neuron Gene Cluster (Fig. 5A) and Muscle Gene Cluster (Fig. 5B), identifying 96 drugs for the muscle cluster and 89 drugs for the motor neuron cluster, with 45 drugs common between the two clusters. The selection criteria for significant drug effects were defined as an FC value greater than 1.5 or less than 0.67. To predict drug-induced fold change, we employed five ML models: Linear Regression, SVM, Random Forest, Gradient Boosting, and Neural Networks. In the motor neuron cluster (Fig. 5A), Gradient Boosting emerged as the top-performing model with the lowest error rate (0.574), followed by SVM with an error rate of 0.581, and Random Forest with 0.605.

Neural Networks and Linear Regression performed less favorably, with error rates of 0.658 and 0.712, respectively. This suggests that Gradient Boosting and SVM are more accurate in predicting the effects of drug candidates on motor neuron gene expression. For the muscle cluster (Fig. 5B), the performance of the models varied depending on the specific drug being analyzed. For example, Random Forest provided the best predictions for nitric oxide production, with a mean error of 0.443. However, for other drugs, Gradient Boosting and Neural Networks often outperformed the other models, highlighting the variability in model performance based on the drug and tissue type.

### Novel Common Repurposed Drugs

To identify novel drug candidates for ALS, a text-mining analysis was performed on the obtained drugs. This analysis revealed five novel candidates: Nilotinib, Trovafloxacin, Apratoxin A, Carboplatin, and Clinafloxacin (Table 1). These five drugs, identified through text-mining analysis, represent promising candidates for repurposing in ALS treatment.

**Table 1:** Text-mining results for drug candidates identified by ML for ALS

Candidate drugs in ALS	# of drug related to articles	# of drug-ALS related to articles	TF	IDF	TF-IDF
Nilotinib	2686	0	0	2.392	0
Trovafloxacin	677	0	0	2.99	0
Apratoxin a	39	0	0	4.23	0
Carboplatin	15,922	0	0	1.619	0
Clinafloxacin	230	0	0	3.459	0
Decitabine	2074	1	0.003	2.504	0.0075
Antimony	7003	2	0.006	1.975	0.0118
Luteolin	6265	2	0.006	2.024	0.0121
Lapatinib	2502	2	0.006	2.422	0.0145
Actinomycin d	14,122	3	0.009	1.671	0.015
Sulforaphane	2522	3	0.009	2.419	0.0217
Hydrocortisone	19,255	5	0.015	1.536	0.0229
Dexamethasone	63,333	11	0.033	1.019	0.0335
Nicotine	43,943	11	0.033	1.178	0.0387
Sevoflurane	10,326	10	0.03	1.807	0.0539
Arachidonic acid	36,896	15	0.045	1.254	0.0561
Pioglitazone	5335	10	0.03	2.094	0.0625
Propofol	23,143	19	0.057	1.456	0.0826
Insulin	361,574	191	0.57	0.263	0.1496
Mercury	43,833	50	0.149	1.179	0.1759

## Discussion

In this study, we applied an integrated network biology and ML-based approach to analyze ALS motor neurons and muscle tissue datasets, aiming to identify key genes and novel repurposed therapeutic candidates. ALS genetic heterogeneity complicates efforts to identify consistent disease markers across tissues (Pansarasa et al., 2018). Studies of motor neuron and muscle tissues have revealed that the genetic alterations contributing to ALS pathogenesis vary between these tissues (Saxena & Caroni, 2011), warranting the separation of datasets into muscle and motor neuron categories.

Using four independent transcriptomic datasets—two for motor neurons and two for muscle tissues—we identified DEGs between ALS patients and healthy controls. It was revealed two significant gene clusters: the Motor Neuron Cluster and the Muscle Cluster, both of which achieved over 60% accuracy in distinguishing between healthy and disease states when analyzed using ML algorithms. Due to the limited availability of transcriptomic datasets for other ALS-affected tissues, we used an independent peripheral blood RNA-seq dataset (GSE234297) for validation. Since peripheral blood represents a different tissue type than motor neurons and muscle tissue, the difference may have seen to the observed decrease in accuracy scores. Several genes identified within the clusters, such as CD44, NDRG2, GSN, and CDK2 in motor neurons (Katzeff et al., 2020, 2020; Matsumoto et al., 2012; Moujalled et al., 2015; Rohm et al., 2019), and PRNP, PABPC1, SPARC, and IRS1 in muscle tissue (Collins et al., 2015; Gao et al., 2023; Halbgebauer et al., 2022; Uozumi et al., 2024), have been previously linked to ALS pathogenesis. These findings reinforce the tissue-specific nature of ALS, where disease mechanisms and molecular pathways may diverge.

In a comprehensive pathway analysis of the gene clusters, several pathways were found to be statistically significant. For muscle tissue, significant disruptions were observed in metabolism and muscle function pathways, with the signal attenuation pathway being the most enriched (q-value = 0.00225; rich factor = 0.2). The pathway is associated with ALS-related neuromuscular junction dysfunction and retrograde signaling deficits observed in preclinical models (Dupuis et al., 2011). Additionally, the insulin-related pathway suggests metabolic disturbances, aligning with reports of altered glucose metabolism in ALS muscle (Lanznaster et al., 2018).

Pathways, such as tyrosine kinase signaling, which regulates muscle repair via IGF-1 (Schiaffino & Mammucari, 2011), and RNA degradation which is linked to TDP-43-mediated RNA processing defects (Buratti et al., 2010), further highlight key mechanisms driving muscle atrophy and dysregulation in ALS. In motor neurons (Fig. 3C), deficits in RET signaling, which impair neurotrophic support (Stansberry & Pierchala, 2023), and ATR activation in response to replication stress, a marker of DNA damage in ALS neurons (Wang et al., 2013), underscore the importance of neuronal survival and DNA repair mechanisms. Enrichment of the p53 signaling pathway and G2/M checkpoints implicates cell cycle dysregulation and apoptosis (Ranganathan & Bowser, 2010), while disrupted neurotrophin signaling (e.g., BDNF/NGF deficits) correlates with motor neuron degeneration (Stansberry & Pierchala, 2023). Collectively, these pathways reflect key hallmarks of ALS, including metabolic dysregulation, RNA/DNA instability, and failed neuroprotection.

Following pathway and gene cluster validation, we identified the Motor Neuron Gene Cluster and Muscle Gene Cluster as key targets for drug repurposing. Using ML models to predict drug-induced fold changes (FCs), we identified several potential drugs. The drugs with the lowest prediction errors were further analyzed using text-mining, which revealed five novel drug candidates: Nilotinib, Trovafloxacin, Apratoxin A, Carboplatin, and Clinafloxacin. Although these agents initially developed for oncology or infectious diseases, they exhibit mechanisms that intersect with ALS pathogenesis.

Nilotinib, a chemotherapeutic tyrosine kinase inhibitor, has demonstrated neuroprotective potential in neurodegenerative disorders such as Parkinson’s disease by promoting autophagy-mediated clearance of protein aggregates (Sachdeva et al., 2024). This mechanism aligns with ALS pathology, where TDP-43 aggregation drives neurodegeneration. However, its broad kinase inhibition raises concerns about off-target effects, particularly in ALS models where kinases such as RIPK1 and EphA4 are implicated in neuroinflammation and axonal degeneration (Imamura et al., 2017). In vitro experimental studies of ALS models are crucial to assess its kinase-specific efficacy and potential toxicity.

Apratoxin A, a natural product isolated from *Moorea producens*, exhibits antitumor activity by inhibiting pathological cell proliferation and protein synthesis. This mechanism may also reduce neuronal degeneration in ALS by potentially decreasing the production of misfolded proteins such as SOD1 or TDP-43 (Nagle et al., 2004). However, its cytotoxicity presents significant challenges for neuronal applications. Dose optimization or the development of structural analogs may be necessary to balance therapeutic efficacy and safety.

Carboplatin, a chemotherapy agent, induces cancer cell death by disrupting DNA repair, which could also modulate neurodegenerative pathways (Kramer et al., 2017). In post-mitotic motor neurons, however, its potential to modulate neurodegenerative pathways—such as by enhancing DNA damage response or synergizing with PARP inhibitors—warrants further investigation, particularly given ALS-associated deficits in DNA repair (Wang et al., 2013).

The fluoroquinolones, Trovafloxacin and Clinafloxacin, emerged as candidates due to their anti-inflammatory properties (Rusu et al., 2023), which may help to mitigate neuroinflammation linked to ALS progression (Zhao et al., 2013). However, fluoroquinolones are known to impair mitochondrial function (Kalghatgi et al., 2013)—a critical concern in ALS, where mitochondrial dysfunction exacerbates motor neuron death (Allen et al., 2019). Rigorous in vivo studies are needed to evaluate their risk–benefit ratio in ALS models.

This study stands out by integrating network biology and ML to identify tissue-specific ALS key genes and drug targets. The dual-cluster approach allowed for a more targeted analysis of ALS-specific mechanisms in motor neurons and muscles, paving the way for more effective therapeutic strategies. Using ML and text-mining techniques to repurpose drugs for ALS presents a novel, scalable approach that could also be applied to other neurodegenerative diseases.

Although the study successfully identified key gene clusters and novel drug candidates, several limitations must be considered. The use of transcriptomic data alone may not capture the full complexity of ALS, which involves proteomic and epigenetic factors. Future research should incorporate multi-omics data for a more comprehensive analysis. Additionally, the novel drug candidates need in vitro and in vivo validation to determine their efficacy in ALS models. Model optimization is another area for improvement, as Gradient Boosting and Random Forest performed better, but further refinement could enhance predictive accuracy.

## Conclusion

This study demonstrates the utility of an integrative bioinformatics and ML approach for identifying novel therapeutic targets and drug candidates for ALS. By focusing on tissue-specific gene clusters, we identified key pathways and five promising drug candidates for future experimental validation. This work highlights the potential for advanced computational methods to drive drug repurposing efforts and supports the development of a network medicine approach for ALS and other neurodegenerative diseases.

## Supplementary Information

Below is the link to the electronic supplementary material.

Supplementary file1 (XLSX 1307 kb)

## Data Availability

The data supporting the findings of this study are publicly accessible in the NCBI GEO database (https://www.ncbi.nlm.nih.gov/geo/) under accession numbers GSE76220, GSE19332, GSE26276, and GSE41414.
